# Interprofessional teaching and learning in the health care professions: A qualitative evaluation of the Robert Bosch Foundation's grant program "Operation Team"

**DOI:** 10.3205/zma001015

**Published:** 2016-04-29

**Authors:** Lukas Nock

**Affiliations:** 1 Institut für Sozialforschung und Sozialwirtschaft e.V. (iso), Saarbrücken, Germany

**Keywords:** interprofessional learning, interprofessional teaching, health care professions

## Abstract

**Aim: **Interprofessional teaching and learning is gaining significance in the health professions. At the same time, the development and implementation of such educational courses is demanding. Focusing on factors critical to success, the aim of this paper is to evaluate the experience gathered by eight grant projects in which interprofessional courses were designed. Emphasis is placed on the level of cooperation between the participating educational institutions, course content, the operative implementation of the course units and their permanent integration into curricula.

**Method: **Data was collected in semi-structured, guideline-based interviews with project leaders and team members (n=43). University and vocational students who had attended the evaluated courses were also included in the survey (n=7) as a means to triangulate data. Analysis was carried out based on qualitative content analysis.

**Results: **A participatory, dialogue-centered model of cooperation appears to be most suited for developing and implementing courses. Belonging to the factors critical to success are the time when courses are offered, the conditions for attendance, the different teaching and learning cultures of the professions involved, preparation and deployment of instructors, and the role played by project coordination. Permanently integrating interprofessional units into medical curricula revealed itself to be difficult.

**Conclusion: **While the development and realization of interprofessional courses can be achieved easily enough in projects, curricular integration of the new course units is challenging. In respect to the latter, not only a large amount of staffing resources and time are required, but also the creation of the necessary system-level structures, not just within the educational institutions (organizational development) but also in the frameworks governing the professions.

## Zusammenfassung

**Zielsetzung:** Interprofessionelles Lehren und Lernen gewinnt in den Gesundheitsberufen zunehmend an Bedeutung. Zugleich gestaltet sich die Entwicklung und Implementierung entsprechender Lehrangebote als voraussetzungsvoll. Ziel der diesem Artikel zugrundeliegenden Evaluation war es, die Umsetzungserfahrungen aus acht geförderten Projekten, in welchen interprofessionelle Lehrangebote entwickelt wurden, mit Blick auf erfolgskritische Faktoren zu untersuchen. Im Fokus standen dabei die Ebene der Kooperation zwischen den beteiligten Ausbildungsinstitutionen, die inhaltliche Ausgestaltung und operative Durchführung der entwickelten Lerneinheiten sowie deren curriculare Einbettung und Verstetigung.

**Methodik: **Die Datenerhebung erfolgte über leitfadengestützte, teilstrukturierte Interviews mit Projektverantwortlichen und -mitarbeitenden (n=43). Zur Datentriangulation wurden außerdem Auszubildende und Studierende, die an den zu evaluierenden Lehrveranstaltungen teilgenommen haben, in die Befragung miteinbezogen (n=7). Die Auswertung erfolgte in Anlehnung an das Verfahren der qualitativen Inhaltsanalyse.

**Ergebnisse: **Für die Entwicklung und Durchführung der Lerneinheiten erscheint ein partizipatives, dialogorientiertes Kooperationsmodell am geeignetsten. Als erfolgskritische Faktoren sind u.a. die zeitliche Ansetzung der Lehrveranstaltungen, ihre Teilnahmebedingungen, unterschiedliche Lehr- und Lernkulturen der beteiligten Professionen, Einsatz und Vorbereitung von Lehrkräften sowie die Rolle der Projektkoordination zu nennen. Schwierig gestaltet sich vor allem die curriculare Einbettung und Verstetigung der interprofessionellen Lerneinheiten in das Medizinstudium.

**Schlussfolgerung: **Während sich die projektförmige Entwicklung und Durchführung interprofessioneller Lehre gut realisieren lässt, gestaltet sich die curriculare Einbettung und Verstetigung der entwickelten Lerneinheiten schwierig. Zu Letzterem bedarf es einerseits größerer personeller und zeitlicher Kapazitäten, andererseits eines Aufbaus förderlicher Strukturen, sowohl innerhalb der Ausbildungsinstitutionen (Organisationsentwicklung) als auch mit Blick auf die (beruf-)politische Rahmensetzung.

## 1. Introduction

The Robert Bosch Foundation is currently funding eight projects involved in developing interprofessional education in the German cities of Berlin (n=2), Bochum, Greifswald, Jena, Lübeck, Mannheim and Munich. The professional socialization of future occupational therapists, midwives, speech therapists, medical doctors, nurses, and physical therapists is being addressed in a targeted manner through the joint development of interprofessional courses at universities and vocational schools. Doing this enables future health care professionals to experience collaboration in a multiprofessional team early on and to acquire the necessary skills and competencies. The project results also serve to anchor interprofessionalism in the standard curricula to sustainably establish the topic in each educational system.

The focus on interprofessionalism zooms in on the problem that interprofessional collaboration has not sufficiently evolved in the German health care system [1], [2], [3], [https://gesellschaft-medizinische-ausbildung.org/aktivitaeten/ausschuesse/interprofessionelle-ausbildung.html accessed on 5 August 2015]. Considering the institutional, structural and legal constitution of the health care system, interlinking the health professions appears very challenging. First of all, there are not only historically based asymmetries between the professions, but also legal ones [4]. This is why physicians have such extensive authority to give orders and delegate tasks in relation to those in the other health professions. At the same time, the division of labor and the hierarchy – particularly in the hospital setting – is dominated by genuine organizing principles for medicine, something that is reflected in the “paramedical” concept of nursing, midwifery, speech therapy, physical and occupational therapy. The tendency to center responsibility on the physician and the predominance of medical culture [4] that are formalized by all this makes multiprofessional dialogue on equal footing difficult to attain. If structurally induced time pressure and a tendency to keep patients in the hospital for ever shorter periods of time increase the communication difficulties between the individual professions in day-to-day hospital operations [5], tendencies to delegate tasks as a means to optimize subsystems [6] encourages the segmentation of the health professions even further.

However, changes can be seen as a result of the professionalization of the occupations (and the associated processes of distinction [7]) that complicate interactive relationships among the professions. Although the academization and professionalization of the health professions may inevitably contribute to improvements in qualifications and to expanding the scope of responsibility and expertise for each profession, targeted and solution-oriented collaboration and communication between the professions is overshadowed by separate agendas, including the promotion of professional self-interests, exclusive claims to particular skills and assertion of superiority on the part of certain professions, and the insistence on having authority [8], [9]. 

The eight model projects, to which the evaluation results refer, all differ in their focus, organization of the interprofessional courses, constellation of the target group, and project architecture. What they do share is a basic understanding of interprofessional learning as a process in which “two or more professions learn with, from and about each other to improve collaboration and the quality of care” [http://caipe.org.uk/about-us/the-definition-and-principles-of-interprofessional-education/ accessed on 5 August 2015].

In the international literature there are numerous references to the effectiveness of interprofessional learning [10], [11], [12], on the challenges and trends seen in the implementation of interprofessional teaching formats and on the various forms of implementation with regard to their advantages, disadvantages, and conditions for implementation [13], [14], [15]. It has been determined that the success of interprofessional teaching is not limited to the educational material imparted or the teaching strategy employed [16]. Just as crucial are the organizational and institutional factors [17]. In the case of the projects evaluated here it is also true that the development and implementation of the interprofessional courses took place in cooperation between independent educational institutions, meaning in an inter-institutional context. In keeping with this, our evaluation focuses on the levels of cooperation between the stakeholders involved in developing and implementing the interprofessional courses, the design of course content, its operative implementation and long-term integration into curricula. The evaluation attempts to identify factors that are critical to successful implementation of interprofessional teaching formats.

## 2. Methods

The methodological approach followed a mixed method design combining qualitative and quantitative methods of social research. The empirical approach first called for document analysis in which all available texts (primarily grant applications and concept papers) were assessed in terms of structure and content for each project. In a second step between February and March, 2015, guideline-based expert interviews (n=43) were conducted with project leaders and team members [18], [19]. The aim was to explore the specific insights of the most important stakeholders (project management/project execution; instructors; evaluators) on a project’s inceptive context, cooperative network, process, organization and content of the course units, along with their estimations of the success and strategies for transfer. A main focus of the expert survey was on the process of project realization, meaning how and with which means the particular project goals (learning outcomes, intension to transfer, etc.) were pursued according to the experts, and which factors turned out to be necessary or limiting in terms of success. These aims formed the main focus of the interview. In parallel to interviewing the experts, semi-structured interviews (n=7) were held with students of medicine, nursing, and therapy who had already taken the interprofessional courses. The primary objective here was to become familiar with the particular course from the perspective of the student, to reconstruct the subjective benefits experienced by individual students [20], [21], and to triangulate the expert view [22]. All interviews were digitally recorded, summarized in full and partially transcribed. Data analysis was carried out based on qualitative content analysis [23]. The analytical rubric was based on both the structure of the interview guidelines (meaning the focal points outlined above) and the dominant interpretations and main topics articulated by those surveyed during the interviews [24], [25]. The results of the qualitative data were compiled into single case studies and also into a comprehensive overview allowing discussion of connections and related contexts, recurring process patterns and influential factors. A final empirical step was begun at the end of 2015 in the form of a standardized student survey of those who had participated in the projects. The collection of this quantitative data was not yet complete at the time this paper was submitted for publication.

## 3. Discussion of the results

The most significant evaluative findings are presented and discussed in the following section. These refer to the organization of the cooperation between the affected educational institutions (3.1), content design (3.2), permanent curricular integration (3.3), and the conducive and restricting factors concerning implementation of the interprofessional courses.

### 3.1. Organization of project cooperation

The development and implementation of interprofessional courses does not simply mean bringing together students of different professions, but rather bringing together, in equal measure, the ideas and approaches of the various people and institutions involved in a project. In terms of the latter, this entails the people and members of a specific profession who are also employees of a particular institution (e.g. a nursing school, medical school, institute for occupational and physical therapy, etc.); as such, these people usually come to the project with two distinct agendas as experts in their fields with their interpersonal skills and as representatives of larger bodies with limited resources, institutional restrictions and overriding interests. Independent of organizational membership is the professional membership of those involved with their specific occupational perspectives, methods of working and interacting which are of importance when confronting challenges such as the ones posed by the projects under evaluation here.

The implied potential for conflict and any requisite need for mediation at the cooperative level were perceived very differently by the project groups. In the following, two typical forms of ideal cooperation emerged to varying degrees in the projects and are compared here:

- **Participatory discursive partnership:** In six of eight cases there was equal cooperation between the participating institutions in which participation by all represented professions/institutions was observed as a guiding principle for all tasks and decision-making. All project phases (conceptual development, grant application, content design/implementation/holding courses and seminars) were accompanied by negotiations between the affected stakeholders. If key persons could be identified in this mode of cooperation, it did not result in predefinition or assignment of roles and functions to the other partners. Rather, the delegation of tasks and the assumption or assignment of responsibility was discursively coordinated within the context of the particular spheres of authority, resources and restrictions. Coordination points were mutually agreed on or jointly delegated.
- **Instrumental partnership:** The starting point in this case is a situation in which the form and content of the project was already defined to a large extent by a main stakeholder prior to the cooperative phase. The instrumental character of this form of cooperation basically lies in the fact that the main stakeholder needs a partner primarily as a means to implement his own ideas, which are not up for discussion. The instrumental nature remains in effect for assigning roles and delegating tasks and responsibilities. In the projects affected by this form of cooperation, instrumental partnerships mostly led to the main stakeholders taking on the tasks relevant to development, while the other partners were primarily occupied with the logistics of recruiting participants from specific professions.

It cannot be stated here which form of cooperation is better. The evaluation showed that none of the eight projects were completely without problems; both modes have advantages and disadvantages. Ideally, cooperation based on dialogue may be for the most part coordinated and result in sustainable decisions, but it can also become a paralyzing model of consensus in the face of limited resources and a lack of pressure to act, or lead to a diffusion of responsibility. Rational decision-making and pragmatism fall under the functional aspect of efficiency as advantages of instrumental cooperation, while the extent to which joint goals can take on a generally binding character in this particular mode of cooperation remains open. 

Still, it is possible to discuss several critical aspects that coincide with a more or less pronounced form of instrumental cooperation:

- **Difficulties in offering courses:** Cancellations due to problems with recruiting students occurred almost exclusively in the projects with instrumental cooperation. It is possible that dialogue-centered cooperation is not only better able to yield an adequate recruiting strategy, but also to acquire, in equal measure, champions and advocates at the participating organizations thereby having a greater effect and generating a higher level of loyalty in the promotion of the courses. Likewise, it can be assumed that a discursive approach is more suitable for doing justice to the diverse organizational features of the participating institutions (e.g. schedules of the academic/vocational programs, different teaching and learning cultures, etc.) in the early phase of project planning.
- **Conformance with traditional role patterns:** With the interaction of the various institutions in mind, it can be stated that the “medical dominance” of the health care system criticized in the discourse surrounding interprofessionalism was not perpetuated in many of the projects, but rather new forms of cooperation, even one on equal footing, were seen. However, there were examples, particularly in connection with more instrumental partnerships, of how a personal determination to accomplish something took a back seat to the determination of a profession to do the same and how the usual hierarchies reasserted themselves in the project’s structure. The descriptions of those surveyed do not paint a picture in which the physicians actively claimed leadership status for themselves. But rather, this was left to the medical schools, whereby the partners from non-medical professions repeatedly cited the reasons for this being the experience and ability of medical doctors to deal with third-party funding and project administration.
- **Conflicts regarding project management and distribution of resources:** Negative developments on the cooperative level were seen in exceptional cases, starting with the participating medical schools. This mainly involved the distribution of resources and opportunities to exercise influence on project management. Such conflicts endangered not only the coming together of the project in the application phase, but also affected the cooperative relationship and made operations difficult.

#### 3.2. Design of course content

Despite the wide variance in course content, meaning the response to precisely how interprofessional learning can be realized or interprofessionalism addressed, two general approaches can be identified; these are outlined in the following along with their implications [16].

- **Thematic intersections as vehicles for interprofessionalism:** What is meant here is the teaching of interprofessionalism using a subject of equal relevance to all the participating professional groups. Ideally, not only knowledge and decision-making skills should be acquired, but learning situations should also be created in which the students can also learn from, about and with each other. In respect to formal integration, this variant provides opportunities for linking the topics of existing curricular units (module combinations) across different study and training programs and the potential for longitudinal integration of multiple units at different points in the curricular programs. This becomes more and more difficult as the number of professions involved increases. In addition, “harmonizing” any asymmetrical knowledge among the professional groups is a challenge.
- **Interprofessionalism as an explicit teaching topic:** In this case, interprofessional collaboration itself is the main focus. To address it, completely new courses were developed, rather than combining existing modules or using common topics covered by different subjects. Naturally, expert input was important to design new courses, the core elements being team conflicts, dealing with stereotypes, becoming familiar with the responsibilities, skills and capabilities, limits and shared goals of the different health professions, communication techniques, informal interaction, etc. This implementation strategy simplified the participation of many professions. In respect to the potential for permanent inclusion or longitudinal integration, however, a sufficient number of interfaces are currently lacking in existing curricula.

Regardless of the strategy chosen, care should be taken to avoid having the content relevant to one profession dominate; an “interprofessional balance” should be reflected in the content of the courses.

#### 3.3. Sustainable curricular integration of the courses

A general problem seen in curricular integration is that not only medical degree programs, but also those of the other health professions, cover an extensive amount of material with individual subjects building off of and recurrently referring to each other over the entire course of study. Restrictions also arise from the rules and regulations governing each institution, such as those affecting training, study, testing, accreditation criteria, and enrollment capacities. Within this context, only one of the eight projects was successful in offering a required course that was mandatory for all of the participating professions. In three additional cases attendance was completely voluntary, whereas hybrid forms were seen in the rest of the projects. Almost all of those surveyed felt integration in the form of a required course was the ideal solution, but this was considered impossible under the existing circumstances by the medical schools. The reasoning behind this rejection most often involved the statute-based enrollment capacities and the much higher numbers of medical students in comparison to the smaller cohorts in the other professions that precludes any feasible interprofessional mix in the courses. Furthermore, scheduling and coordinating suitable periods of time within the various curricula proved to be a great barrier. Ultimately, those surveyed at the medical schools saw the introduction of a required interprofessional module as involving the impossibility of making compromises or cutting other curricular content, thus potentially affecting satisfaction of the medical licensing requirements.

The experts interviewed were unable to say much about the continuation of the interprofessional courses at the time the interviews were conducted. This is due in part to the fact the projects are relatively new and the need for intensive coordination and staffing. Above all, the issue of continuation is constrained by legal restrictions (licensing requirements, rules and regulations governing studies and testing, enrollment capacities), organizational and logistical challenges (differing schedules of different educational programs; different cohort sizes), and many other system-level barriers. At the administrative level, at least, long-term inclusion of interprofessional courses appears to be less of a challenge for the participating vocational schools where, despite state-defined curricula and legislation governing vocational training, administrators have both sufficient authority and the necessary leeway to make decisions about implementing changes at their institutions. The medical schools and university teaching hospitals differ from vocational schools in that they are much larger organizations with broader and deeper hierarchies of management. As academic institutions they also have a highly differentiated system of departments, are subdivided into numerous smaller units, and have complex decision-making processes in contrast to other post-secondary educational institutions.

#### 3.4. Conducive and restricting factors

In the overall consideration of the projects, several factors can be identified as hindering or facilitating planning, implementation and offering the courses.

- **Course attendance**: Over the course of several projects it became clear that interprofessional courses can be negatively impacted if the joint learning sessions are offered on a voluntary basis for medical students but are mandatory for nursing students. The negative influence of differing attendance rules may especially play a role if the participating groups have not been informed about the particular context for this.
- **Time point of the courses:** When interprofessional courses can best be offered during training or education cannot be answered conclusively by the data collected for this paper. However, it was clear that scheduling courses in close proximity to exams was disadvantageous in terms of recruiting participants if the interprofessional courses did not cover exam-relevant content. Scheduling courses early or late in the day can also be viewed as a hindering factor, especially if participants have long distances to travel in order to attend.
- **Different “learning cultures”:** Different cultures of learning appear to play a considerable role in connection with the willingness to attend extra-curricular courses. This appears to have less to do with specific professions than with the socialization within an academic versus non-academic educational system. While vocational students find themselves in a highly defined and tightly focused educational system with alternating practical units, university students are more strongly required (and as a consequence much more used) to taking charge of their own education.
- **Teaching staff:** As direct conveyors of interprofessional learning content, instructors are the most important point of transmission between the conceptual levels of the project and its operative goals. Depending on the context, instructors were placed more in the role of moderator or more in that of classic teacher; they participated to varying degrees in designing the content of the units including involvement in the contextual inception of the project. In the expert interviews interprofessional teaching was repeatedly appraised as very demanding and the importance of extensive preparation prior to teaching was emphasized.
- **Scheduling flexibility of the institutions:** Finding suitable time slots within the curricula of the participating institutions revealed itself to be the single largest challenge for all of the projects. The degree of complexity increased in proportion to the number of professions involved. In connection with these coordination challenges, flexibility regarding scheduling is a crucial aspect for enabling organization.
- **Project coordination:** In all of the projects, coordinators fulfilled a central function connecting the operative and strategic levels and served as a contact point for the interfaces at the participating institutions. The administrative efforts associated with joint collaboratives between institutions (especially if pilot courses are involved) require appropriate staffing.

The results of international studies confirm our findings and indicate the presence of further influential factors, primarily affecting learning outcome. Focus is on the importance of small-group work, a clearly articulated teaching strategy, an unthreatening learning environment, and enabling reflection on practical experiences [16].

## 4. Conclusion

Considered as a whole, these eight projects clearly demonstrate the commitment and wealth of ideas with which interprofessional learning can be realized in a diversity of teaching formats. At the same time, the evaluation showed the effort connected with interprofessional teaching in the health professions and which organizational, institutional and legal barriers make its implementation so difficult, particularly in terms of longitudinal curricular integration. As a result, it was only possible in individual cases to implement curricular courses that addressed all students of the particular participating professions with the same degree of commitment.

Without a doubt, the post-secondary vocational institutions and very specifically the university medical schools play a key role in the permanent implementation of interprofessional courses [16], [17], [26]. Appropriate staffing and other resources are necessary in order to realize these concepts as overall concepts and not just as isolated modules taught by individual instructors [26]. However, educational institutions must open up to the idea of organizational development and establish structures and positions of authority to implement interprofessionalism as a shared, long-term goal. It is precisely the great relevance given to interprofessionalism in health care policy and the discourse among experts in the health professions that make voluntary extra-curricular courses seem a very flimsy solution. In fact, it is doubtful that interprofessional learning will be given any sort of appropriate institutional expression if it is seen as being a matter of student self-regulation or the commitment of individual teachers.

Overall, sustained integration of interprofessional courses does seem to depend solely on the effort of the project leaders and coordinators, but is just as dependent on higher hierarchical levels and decision-making bodies (professional associations, scientific societies, university administration, stakeholders in health care policy) that were not involved within the scope of these grant projects. At the political level frameworks must be negotiated that not only compel educational institutions to permanently implement interprofessional courses into curricula, but also ensure the provision of the resources and authority necessary to accomplish this. Within the context of the evaluation results seen here this appears to represent the greatest need for action in medical curriculum development, specifically granting interprofessional learning a lasting place in medical education.

Approval of the position paper of the GMA Committee on Interprofessional Education in the Health Professions [26] and the National Competency-based Catalogue of Learning Objectives for Undergraduate Medical Education by the Medizinischer Fakultätentag [http://www.nklm.de] represent positive political signals for establishing structures encouraging innovation in and development of interprofessional teaching in the health professions.

## Acknowledgement

We wish to express our gratitude to all the project participants for their willingness to share their experiences in the interviews.

## Competing interests

The author declares that he has no competing interests.
